# Ethical and Legal Challenges in Caring for Older Adults with Multimorbidities: Best Practices for Nurses

**DOI:** 10.3390/healthcare12161585

**Published:** 2024-08-09

**Authors:** Abdulaziz M. Alodhialah, Ashwaq A. Almutairi, Mohammed Almutairi

**Affiliations:** 1 Department of Medical Surgical Nursing, College of Nursing, King Saud University, Riyadh 11451, Saudi Arabia; mohalmutairi@ksu.edu.sa; 2 School of Nursing & Midwifery, Monash University, Melbourne, VIC 3800, Australia; ashwaq.almutairi@monash.edu

**Keywords:** ethical challenges, legal challenges, multimorbidity, nursing, older adults, health outcomes

## Abstract

This study explores the ethical and legal challenges faced by nurses in caring for older adults with multimorbidities, focusing on issues related to patient autonomy, polypharmacy, and end-of-life care. Through semi-structured interviews with 15 nurses in Riyadh, Saudi Arabia, the research highlights the complexities of obtaining informed consent from patients with cognitive impairments and the ethical dilemmas of balancing autonomy and safety. The management of polypharmacy emerged as a significant concern, with nurses emphasizing the importance of thorough documentation and coordination among healthcare providers to prevent adverse drug interactions. End-of-life care and advance directives posed further challenges, particularly when family members disagreed with patients’ documented wishes. The study underscores the need for comprehensive strategies, including ongoing education, clear communication, and supportive institutional policies, to address these issues effectively. The findings suggest that enhancing nurses’ understanding of ethical principles and legal requirements is crucial for improving patient care and ensuring compliance with regulatory standards. Future research should aim to develop evidence-based guidelines to support nursing practice in managing these ethical and legal challenges.

## 1. Introduction

The aging population globally is experiencing an unprecedented increase, leading to a rise in the prevalence of multimorbidity, defined as the coexistence of two or more chronic conditions within an individual [1,2]. This phenomenon is particularly pronounced among older adults, who are often confronted with a complex interplay of health issues that significantly affect their quality of life and healthcare needs [3]. As the demographic shift continues, the ethical challenges associated with providing care to this population have become more prominent, necessitating a comprehensive understanding and proactive management by healthcare professionals, particularly nurses [4,5].

Multimorbidity in older adults is associated with numerous challenges, including polypharmacy, frequent hospitalizations, and increased healthcare costs [6,7]. These individuals often require intricate care plans that involve multiple healthcare providers, making coordination of care crucial [8]. The prevalence of multimorbidity increases with age, with more than 65% of individuals aged 65 and older experiencing two or more chronic conditions. This demographic trend underscores the need for specialized care strategies that address both the medical and psychosocial aspects of aging [9].

One of the primary ethical challenges in caring for older adults with multimorbidities is maintaining patient autonomy [10]. Respect for autonomy is a fundamental ethical principle in healthcare, emphasizing the patient’s right to make informed decisions about their care [11]. However, cognitive impairments, common among older adults, can complicate decision-making processes. So, nurses caring for older adults must balance respecting autonomy with ensuring that patients are not exposed to harm due to their impaired decision-making abilities [12].

The principle of beneficence, which obligates healthcare providers to act in the best interest of the patient, often intersects with ethical dilemmas in this context [13]. Nurses frequently encounter situations where the best medical intervention may not align with the patient’s or their family’s wishes [14]. Aggressive treatments that extend life may not necessarily improve the quality of life for older adults with multiple chronic conditions [15]. There is a growing recognition of the need for palliative care approaches that focus on comfort and quality of life rather than merely extending life [16].

Moreover, the principle of non-maleficence, which mandates that healthcare providers do no harm, is critically important in the management of multimorbid older adults. Polypharmacy, the concurrent use of multiple medications, is a common issue that can lead to adverse drug reactions and interactions, posing significant risks to patients [17]. A study highlights that polypharmacy is associated with increased morbidity and mortality among older adults, necessitating careful medication management and regular review by healthcare professionals [18].

According to the American Nurses Association (2015), documentation serves as a critical component of patient care, providing a detailed account of the patient’s condition, the care provided, and the rationale for clinical decisions [19]. Issues can arise if documentation is incomplete or inaccurate, potentially leading to adverse outcomes for patients and ramifications for healthcare providers [20].

Patient rights, including the right to informed consent and the right to refuse treatment, are legally protected and must be upheld by healthcare providers [21]. The concept of informed consent is legally binding and requires that patients receive comprehensive information about their diagnosis, treatment options, and potential risks and benefits [22]. However, obtaining informed consent from older adults with cognitive impairments presents unique challenges, healthcare providers must use clear communication strategies and, when necessary, involve family members or legal representatives to ensure that the patient’s rights are respected [23].

Furthermore, the issue of advance directives and end-of-life care preferences adds another layer of complexity to the ethical landscape [24]. Advance directives, including living wills and durable powers of attorney for healthcare, allow individuals to articulate their preferences for medical treatment in the event that they become incapacitated [25]. Advance directives are underutilized, and many patients’ end-of-life care preferences are not documented or honored, leading to ethical dilemmas for healthcare providers [26].

Nurses play a pivotal role in advocating for the needs and preferences of older adults with multimorbidities [27]. Advocacy involves not only supporting patients’ rights and autonomy but also addressing systemic issues within the healthcare system that may impede the delivery of high-quality care [28]. As patient advocates, nurses must be knowledgeable about ethical principles and requirements and be prepared to navigate complex situations that arise in the care of this vulnerable population [29].

The increasing prevalence of multimorbidity among older adults presents significant ethical and legal challenges for healthcare providers, particularly nurses [30]. These challenges include maintaining patient autonomy, ensuring beneficence and non-maleficence, managing polypharmacy, and navigating complex legal requirements related to documentation, informed consent, and advance directives [31]. Addressing these issues requires a comprehensive approach that integrates ethical principles with legal standards and best practices. By doing so, nurses can provide compassionate, patient-centered care that respects the rights and dignity of older adults with multimorbidities.

### 1.1. Aim of the Study

The aim of this study is to explore and understand the ethical and legal challenges faced by nurses in the care of older adults with multimorbidities. It seeks to identify best practices that can be implemented to address these challenges, ensuring that nursing care is both ethically sound and legally compliant. By gaining insights into the experiences and perspectives of nurses, this study aims to contribute to the development of guidelines and strategies that enhance the quality of care for this vulnerable population.

#### Research Questions

1. What are the primary ethical challenges encountered by nurses when providing care to older adults with multimorbidities, and how do they navigate these challenges in their daily practice?
2. What are the legal issues faced by nurses in the care of older adults with multimorbidities, and what best practices can be identified to ensure compliance with legal standards while maintaining high-quality patient care?

## 2. Materials and Methods

### 2.1. Study Design and Participants

This study employed a qualitative descriptive design, deeply rooted in the epistemological frameworks of naturalism and constructivism. Naturalism posits that realities are multiple and subjective, while constructivism emphasizes the interaction between the researcher and the subject in shaping the findings. These frameworks were chosen for their ability to capture the complex, lived experiences and nuanced interactions between healthcare professionals and patients regarding ethical and legal challenges in caring for older adults with Multimorbidities.

To ensure methodological rigor and transparency, our approach adhered closely to the Standards for Reporting Qualitative Research (SRQR) guidelines. This adherence facilitated a systematic and reflective inquiry into the ethical and legal challenges faced by nurses, allowing for detailed exploration and credible documentation of emergent themes that authentically represent participants’ experiences. By integrating these epistemological principles, we aimed to illuminate the subjective and often tacit knowledge that informs nursing practice in this critical area [32].

### 2.2. Setting

The study was conducted from January 2024 to March 2024 in various healthcare facilities in the Riyadh region of Saudi Arabia. Riyadh, the capital city, is known for its advanced healthcare infrastructure and diverse population, providing a rich environment for exploring the complexities of nursing care for older adults with multiple chronic conditions. The healthcare facilities were selected based on their high volume of older adult patients and their comprehensive nature of services, including chronic condition management, acute care, and palliative care. This selection ensured a diverse and representative sample of nursing practices and patient interactions.

### 2.3. Recruitment and Sampling

A purposeful sampling technique was utilized to recruit 15 registered nurses actively practicing in the Riyadh region. Participants were selected based on their experience in caring for older adults with multimorbidities, ensuring a comprehensive understanding of the ethical and legal challenges in this context.

**The inclusion criteria were as follows:**

- Registered nurses aged 23 years or older.
- A minimum of 1 year of experience in nursing, specifically in environments where they regularly care for older adults with multimorbidities.
- Willingness to participate and provide informed consent.

**The exclusion criteria were as follows:**

- Nurses working in fields other than those involving direct patient care of older adults with Multimorbidities.

The rationale for the sample size of 15 nurses was based on achieving data saturation, a point where no new themes or insights emerge from additional data collection. This sample size was deemed sufficient to capture diverse perspectives and experiences while allowing for in-depth exploration of the research questions. The participants’ ages ranged from 26 to 55 years, with varying levels of educational background from diplomas to master’s degrees in nursing. This diversity in experience and education provided a broad range of insights into the challenges and best practices in nursing care for older adults.

### 2.4. Development of Interview Guide

The development of the interview guide was a meticulous process influenced by established frameworks and prior studies that explored ethical and legal challenges in nursing [33,34,35]. The guide drew upon foundational works on ethical principles and legal requirements in healthcare. A preliminary literature review identified gaps in existing studies, particularly focusing on the unique challenges faced by nurses in Saudi Arabia. The guide was reviewed by experts in qualitative research and nursing ethics, and piloted with a small group of nurses to ensure clarity and relevance. Questions were formulated to explore themes such as the challenges in obtaining informed consent, managing polypharmacy, and navigating end-of-life care decisions.

### 2.5. Data Collection

Data collection for this study was conducted from January 2024 to March 2024, using a combination of face-to-face and telephone interviews, observations, and document reviews to gather comprehensive and multifaceted insights into the ethical and legal challenges faced by nurses in caring for older adults with Multimorbidities.

The primary method of data collection was semi-structured interviews. Each interview lasted approximately 45 to 60 min and was conducted in a private room within the healthcare facilities to ensure a conducive environment for open and honest discussion. Some interviews were conducted face-to-face, allowing for richer interactions and the observation of non-verbal cues, which provided additional context to the verbal responses. To accommodate the busy schedules and preferences of some nurses, telephone interviews were also utilized. While these lacked the advantage of observing non-verbal communication, they offered greater flexibility and enabled participation from nurses who might otherwise have been unavailable. Interviews were conducted in both Arabic and English, depending on the participants’ preferences. Bilingual experts were involved in the interviews to ensure accurate translation and to maintain the integrity of the data. The interview guide was designed to explore themes such as challenges in obtaining informed consent, managing polypharmacy, and navigating end-of-life care decisions.

In addition to interviews, observational data were collected to provide a real-time perspective on the ethical and legal challenges in nursing practice. Structured forms were used to systematically record observations of nurse–patient interactions, focusing on non-verbal communication, practical applications of ethical and legal principles, and immediate responses to patient needs. These observations were conducted in various settings within the healthcare facilities, including patient rooms, nurse stations, and common areas, to capture a broad spectrum of interactions and practices.

Document reviews complemented the interviews and observations by providing institutional and procedural context. Relevant documents such as nursing reports, care guidelines, and institutional policies were reviewed to understand the framework within which nurses operate. These documents offered insights into the standard practices, protocols, and policies that guide nursing care for older adults with multimorbidities.

Throughout the data collection process, meticulous attention was given to capturing detailed and nuanced information. Immediate reflections and non-verbal cues were noted during and after the interviews and observations, enriching the verbal data and providing a deeper understanding of the participants’ experiences. All interviews and observations were audio-recorded, with participants’ consent, to ensure accurate and comprehensive data capture. These recordings were later transcribed verbatim for detailed analysis.

### 2.6. Credibility of the Study

The study incorporated semi-structured interviews, document analysis, and observational data. The semi-structured interviews with 15 registered nurses provided rich qualitative insights into their experiences and strategies in managing ethical and legal challenges while caring for older adults with multimorbidities. These interviews formed the core data source, offering deep and nuanced perspectives directly from the practitioners.

In addition to interviews, document analysis was conducted on relevant materials such as nursing reports, care guidelines, and institutional policies. This analysis provided a broader context and allowed for cross-verification of the qualitative data obtained from the interviews. The documents helped to corroborate participants’ accounts and highlighted institutional support and constraints related to ethical and legal nursing practices.

Observational data further enriched the study by capturing real-time nurse–patient interactions in healthcare settings. Structured forms were used during observations to systematically record behaviors and interactions, ensuring consistency and reliability in data collection. These observations provided an experiential dimension to the study, offering concrete examples of how ethical and legal principles are applied in practice and validating the themes emerging from the interviews and document analysis.

To enhance the validity of the findings, several methodological strategies were employed, which involved systematically cross-verifying data from interviews, document analysis, and observations to ensure consistency and robustness. This process helped to build a comprehensive and reliable understanding of the ethical and legal challenges faced by nurses. Collaborative analysis was another crucial component, where multiple researchers participated in data collection and analysis. This approach minimized potential biases and allowed for a more nuanced interpretation of the data, as diverse perspectives were considered.

Regular peer debriefing sessions were conducted with the research team to review and validate emerging themes and findings. These sessions provided a platform for critical examination and reflection on the data, ensuring that the analysis was thorough and accurate. The iterative process of peer debriefing helped to refine the themes and ensure that they authentically represented the participants’ experiences.

Member checking was also employed to further validate the findings. Summaries of the initial themes were shared with participants to verify the accuracy and completeness of the interpretations. Participants were invited to provide feedback, ensuring that their perspectives were faithfully represented and any discrepancies or misunderstandings were addressed.

### 2.7. Data Analysis

Data analysis followed a thematic approach as outlined by Braun and Clarke (2006), involving six detailed phases to ensure a rigorous and comprehensive understanding of the data. This approach allowed for the systematic identification, analysis, and reporting of patterns within the data, providing rich and nuanced insights into the ethical and legal challenges faced by nurses caring for older adults with multimorbidities [36].

1. **Familiarization with the Data**:
  - **Initial Immersion**: Researchers began by reading and re-reading the interview transcripts to immerse themselves thoroughly in the content. This initial immersion helped researchers gain a deep understanding of the context and nuances of each participant’s experiences.
  - **Transcription**: Verbatim transcriptions of the interviews were generated by two trained investigators to ensure accuracy. Interviews conducted in Arabic were simultaneously translated into English by investigators fluent in both languages. An independent research assistant cross-verified selected English transcripts with their Arabic counterparts for accuracy.
  - **Initial Notes**: During the familiarization phase, researchers made initial notes and observations about potential patterns, significant statements, and emerging themes.
2. **Generating Initial Codes**:
  - **Systematic Coding**: Initial codes were generated systematically across the entire dataset. Researchers identified recurring themes and patterns in the data, focusing on meaningful segments of text that captured key aspects of the participants’ experiences and perceptions.
  - **Collaborative Effort**: The coding process was collaborative, involving discussions among the research team to refine and validate the initial codes. This collaboration ensured that the codes accurately reflected the data and minimized individual biases.
3. **Searching for Themes**:
  - **Organizing Codes**: The generated codes were then organized into potential themes. This phase involved grouping related codes together to form broader patterns and relationships, which encapsulated significant aspects of the data.
  - **Preliminary Themes**: The research team identified several preliminary themes that represented the recurring ideas and concepts within the data. These preliminary themes were discussed and refined to ensure they were comprehensive and reflective of the participants’ experiences.
4. **Reviewing Themes**:
  - **Refinement Process**: The identified themes were reviewed and refined by the research team to ensure they accurately represented the data. This process involved checking if the themes worked in relation to the coded extracts and the entire dataset.
  - **Consensus Building**: The team engaged in collaborative discussions to resolve any discrepancies in theme identification and refinement, fostering a consensus-driven approach. Themes that did not have enough supporting data were discarded or merged with other themes.
5. **Defining and Naming Themes**:
  - **Detailed Definitions**: Each theme was defined and named to clearly convey its essence and relevance to the research questions. Detailed definitions and descriptions were developed for each theme, highlighting the core concepts and insights derived from the data.
  - **Sub-Themes**: Where applicable, sub-themes were identified to capture more specific aspects of the broader themes, providing a nuanced understanding of the data.
6. **Producing the Report**:
  - **Integration and Reporting**: The final phase involved producing a comprehensive report of the findings. The report integrated the themes into a coherent narrative that addressed the research objectives.
  - **Illustrative Quotes**: The report included direct quotes from participants to illustrate and support the themes, ensuring that the voices of the nurses were authentically represented.
  - **Implications and Conclusions**: Key findings and their implications were highlighted, providing insights into the ethical and legal challenges in nursing care for older adults with multimorbidities.

Throughout the thematic analysis, an iterative process was employed to enhance the rigor and validity of the findings. Several researchers participated in theme identification, which helped in triangulating the findings and ensuring a comprehensive and nuanced understanding of the data. Investigator validity was crucial in minimizing potential biases and providing multiple perspectives on the data, thereby strengthening the results.

### 2.8. Ethical Considerations

The study was conducted in accordance with the ethical principles outlined in the Declaration of Helsinki, and approved by the ethical committee of King Saud University (KSU_HE-24-002). Informed consent was obtained from all participants prior to their inclusion in the study. Participants were informed about the study’s aims, procedures, potential risks, and benefits. Confidentiality and anonymity were assured, with data stored securely and accessible only to the research team.

## 3. Results

### 3.1. Participant Characteristics

Table 1 provides an overview of the 15 nurses who participated in the study. The participants exhibited a range of nursing experiences, with years of practice varying from 2 to 30 years. This spectrum includes both relatively new graduates and highly experienced practitioners, offering diverse perspectives on the ethical and legal challenges in caring for older adults with Multimorbidities. The participants’ ages range from 26 to 55 years and span different stages of adult life, indicating potential generational differences in their views on nursing practices and care delivery. The majority of the participants are female, consistent with the long-standing prevalence of women in the nursing profession. This gender distribution reflects the broader demographics of the nursing workforce. This spectrum of ages, educational levels, and years of experience (ranging from 2 to 30 years) provided a broad perspective on the ethical and legal challenges faced in caring for older adults with Multimorbidities.

### 3.2. Thematic Analysis

Table 2 elaborates on the three themes identified through the thematic analysis, each reflecting the ethical and legal challenges faced by nurses in caring for older adults with Multimorbidities.

**I.**  **Ethical Dilemmas in Patient Autonomy and Consent**

**Challenges in Obtaining Informed Consent and Decision-Making with Cognitive Impairment:**

Nurses face significant difficulties in obtaining informed consent from elderly patients with cognitive impairments, often involving family members for clarity. P#3 noted, “It’s often difficult to ensure that patients fully understand the information we provide due to their cognitive limitations”. P#10 added, “We spend extra time explaining and sometimes involve family members, but even then, it’s not always clear if the patient truly grasps the situation”. P#7 highlighted, “We want to honor their wishes, but sometimes it’s a fine line between respecting their autonomy and protecting them from harm”.

Ethical dilemmas arise when patients seem confused despite explanations, as P#5 shared, “In one case, despite multiple attempts to explain the treatment options, the patient still seemed confused. We had to rely on the family to make the final decision”. P#11 added, “There are times when a patient agrees to a procedure just to please their family, without fully understanding the implications”.

Regular challenges occur when patients’ cognitive limitations impede their decision-making. P#1 noted, “When people with dementia refuse therapy, we face potential ethical dilemmas”. P#5 mentioned, “We often navigate ethical gray areas where the patient’s decision-making capacity is compromised, requiring a sensitive and patient-centered approach”.

P#13 shared, “It’s heartbreaking when a patient doesn’t understand their condition and refuses necessary treatment. We have to consider their past wishes, family input, and medical best practices”. P#9 explained, “We work closely with family members to make decisions aligning with the patient’s known preferences and values”. P#7 added, “We sometimes involve the ethics committee to help resolve particularly difficult cases, ensuring all legal and ethical aspects are considered”.

**Balancing Autonomy and Safety:**

Nurses underlined the significance of striking a balance between respecting patient autonomy and ensuring patient safety. P#6 went on to say, “There are times when we have to make tough calls to protect the patient, even if it means going against their immediate wishes. This is particularly challenging when legal and ethical guidelines are in tension, and the nurse must prioritize patient safety while still honoring the patient’s autonomy as much as possible”. P#14 said, “We strive to involve patients in their care decisions as much as possible, but safety must always be a priority. In situations where patients’ decisions might put them at significant risk, we must navigate the legal implications of overriding their choices, which requires thorough documentation and adherence to institutional policies”.

P#2 highlighted, “Ensuring safety while respecting autonomy is one of the hardest parts of our job, especially with patients who have fluctuating cognitive abilities. The legal responsibility to protect these patients can sometimes necessitate interventions that limit their autonomy, which we handle with great care and clear communication to ensure that both ethical and legal standards are upheld”. P#9 shared, “We had a patient with severe dementia who insisted on leaving the hospital against medical advice. While respecting his autonomy was important, his condition posed a significant risk to his safety. We had to involve his family and legal advisors to make an informed decision that balanced his rights with his well-being”. P#11 added, “There was a case where a diabetic patient refused insulin because of fear of needles. We respected her autonomy but also had to address the imminent risk to her health. We collaborated with her physician to explore alternative administration methods and provided extensive education and support to alleviate her fears while ensuring she received the necessary treatment”.

**II.**  **Managing Polypharmacy and Patient Safety**

**Risks of Adverse Drug Reactions:**

Polypharmacy has arisen as a major ethical and legal problem. Nurses noted difficulty managing several prescriptions for patients with multimorbidities, emphasizing the dangers of harmful drug interactions. P#12 said, “The risk of adverse drug interactions is high, and we have to be very vigilant in monitoring their medication regimens”.

P#8 highlighted, “It’s a constant challenge to balance the benefits and risks of multiple medications, especially when patients are seeing multiple specialists who might not always communicate effectively. As nurses, we are responsible for keeping track of all medications a patient is taking, educating patients about potential interactions, and ensuring adherence to their medication schedules”. P#4 explained, “We often see patients with prescriptions from different doctors, and it’s our job to ensure that these don’t conflict. While doctors prescribe medications based on their specialty and the patient’s specific conditions, it falls upon us as nurses to oversee the comprehensive medication management. This includes coordinating with various doctors to clarify prescriptions, identifying potential drug interactions, and making sure that any changes in medication are communicated and understood by all parties involved”.

**Documentation and Regular Reviews:**

Participants underlined the need for accurate documentation and regular drug evaluations to guarantee patient safety. According to P#9, “Accurate documentation is crucial to track all medications a patient is on, which helps in identifying potential interactions”. P#14 stated, “Regular medication reviews are essential to adjust treatment plans and minimize risks, but they require time and coordination among the healthcare team”. P#11 stated, “We need to document every medication change meticulously to avoid errors and ensure continuity of care”.

**Coordination Among Healthcare Providers:**

Effective communication and cooperation among healthcare practitioners were identified as critical to managing polypharmacy safely. P#15 states, “Coordination with other healthcare professionals is critical to managing polypharmacy. We need to guarantee that everyone is on the same page about the patient’s pharmaceutical regimen”.

P#7 added, “Regular team meetings help us discuss and update each patient’s medication regimen, but it’s challenging to keep everyone informed all the time. There are instances where changes made by one specialist are not communicated to others, leading to potential risks for the patient”. P#3 stated, “Having a centralized system for medication records would greatly improve our ability to manage polypharmacy effectively. It would ensure that all healthcare providers have access to the most current and complete medication information, reducing the risk of adverse drug interactions”. P#10 highlighted another aspect, “In our facility, we sometimes use telehealth consultations to bridge the communication gap between different specialists. This helps us coordinate better, especially for patients who are unable to visit multiple doctors frequently”.

P#5 provided an example, “We had a patient who was prescribed conflicting medications by two different specialists. By maintaining open communication channels and conducting a thorough medication review during a team meeting, we were able to identify the conflict and adjust the regimen accordingly”. P#11 emphasized the importance of patient involvement, “We also educate patients and their families to keep an updated list of all medications and to bring this list to every appointment. This empowers patients to be active participants in their care and helps us in maintaining an accurate and safe medication regimen”.

**III.**  **End-of-Life Care and Advance Directives**

**Honoring Advance Directives:**

Nurses highlighted the importance of honoring advance directives to respect patient autonomy and ensure appropriate end-of-life care. P#15 stated, “It’s crucial to have clear communication with patients and their families about their wishes for end-of-life care. We need to ensure that these directives are documented and accessible to all healthcare providers involved”.

P#7 explained, “Regular team meetings help us discuss and update each patient’s care plan, including their advance directives. However, it can be challenging to keep everyone informed all the time”. Effective communication and coordination among healthcare practitioners are essential to ensure that the patient’s wishes are respected.

P#3 emphasized, “Having a centralized system for advance directives would greatly improve our ability to honor patients’ end-of-life wishes. It would ensure that all healthcare providers are aware of and can adhere to the patient’s directives, reducing the likelihood of conflicts or misunderstandings”.

**Difficult Conversations:**

It was brought to the attention of the participants that discussions regarding advance directives and choices regarding end-of-life care are frequently difficult but essential to delivering adequate care. P#2 stated, “These conversations are tough, but they’re essential to ensure that we respect the patient’s wishes and provide care that’s aligned with their values”. P#6 agreed with this point of view, saying, “It’s our responsibility to initiate these discussions and ensure that patients and their families understand the implications of their choices. Often, legal and ethical guidelines require us to document these conversations meticulously to avoid any misunderstandings later”. P#5 concluded by saying, “We try to approach these topics sensitively, but it’s always a delicate balance. We must ensure the patient’s autonomy is respected while also considering their family’s perspectives and the potential emotional burden”. P#10 added, “One of the biggest challenges is explaining the medical and legal ramifications of their choices in a way that’s understandable and compassionate. Patients and families often have differing opinions, and we need to mediate these discussions to find a common ground”. P#7 shared an example, “I had a patient whose family wanted to pursue aggressive treatment, but the patient had expressed a desire for palliative care only. Navigating these conflicting wishes required careful communication and a thorough understanding of both the ethical implications and legal requirements”. P#3 emphasized the importance of continuous education, “We regularly update our training on how to handle these conversations, including understanding cultural sensitivities and legal aspects. This helps us feel more prepared and confident in these challenging situations”.

**Legal Implications of End-of-Life Decisions:**

The legal repercussions that may result from decisions about end-of-life care were another major source of anxiety for nurses. P#15 observed, “We need to be very careful. Any deviation from the documented desires of the patient can lead to legal penalties”. This highlights the importance of adhering strictly to the patient’s advance directives to avoid potential legal consequences. P#9 emphasized, “Clear and precise documentation of advance directives helps protect both the patient’s rights and the healthcare providers. Without proper documentation, we risk legal action from family members who might disagree with the decisions made”. Nurses often find themselves navigating complex legal terrain, ensuring that the patient’s wishes are followed while also protecting themselves and their colleagues from legal repercussions. P#11 added, “Understanding the legal framework surrounding end-of-life care is essential to avoid potential legal issues. We frequently consult with legal advisors and ethics committees to ensure that our actions are in line with both the patient’s wishes and legal requirements”. This underscores the necessity for nurses to be well versed in legal protocols and to maintain meticulous records.

P#7 provided an example, “We had a case where the family wanted to continue life-sustaining treatment despite the patient’s advance directive stating otherwise. It was a very challenging situation, but by following the legal documentation and involving the hospital’s legal team, we were able to honor the patient’s wishes without facing legal consequences”. Another example from P#3 illustrated, “In one instance, a patient had not updated their advance directive, and there was confusion about their current wishes. This led to a legal dispute among family members. We had to involve social workers, legal advisors, and even a court ruling to resolve the issue, which highlighted the critical need for clear and current documentation”.

In addition to interviews, observational data were collected to provide real-time perspectives on the ethical and legal challenges in nursing practice. Structured forms were used to systematically record observations of nurse–patient interactions, focusing on non-verbal communication, practical applications of ethical and legal principles, and immediate responses to patient needs. Document reviews provided institutional and procedural context, offering insights into the standard practices, protocols, and policies that guide nursing care for older adults with multimorbidities.

## 4. Discussion

The findings of this study highlight the multifaceted ethical and legal challenges that nurses face in the care of older adults with multimorbidities. These challenges revolve around patient autonomy, polypharmacy, and end-of-life care, reflecting the complex nature of nursing in this context.

The ethical dilemmas associated with patient autonomy and informed consent are particularly pronounced. Nurses frequently encounter situations where older adults, especially those with cognitive impairments, are unable to fully comprehend their treatment options. This issue is compounded by the need to respect patient autonomy while ensuring their safety. Previous studies have shown that cognitive impairments can significantly hinder the decision-making process, necessitating a delicate balance between autonomy and beneficence [11,37]. The necessity for family involvement in decision-making processes is crucial, as family members often act as surrogates in the consent process [38]. This dynamic can create tension, especially when the patient’s wishes conflict with those of the family, or when the patient’s ability to understand and make decisions fluctuates [39].

Informed consent is a cornerstone of ethical medical practice, yet it becomes problematic when patients are cognitively impaired [40]. Research suggests that a significant proportion of older adults with dementia are unable to provide fully informed consent, raising ethical concerns about autonomy and decision-making capacity [41,42]. Nurses often have to balance the legal requirements for informed consent with the practical realities of cognitive impairment, which can lead to ethical dilemmas and require sensitive handling [43]. Additionally, the involvement of family members as surrogates can both aid and complicate the decision-making process. Family members may have differing views on the best course of action, and these views may not always align with the patient’s wishes or best interests [44].

Polypharmacy represents another significant challenge. The concurrent use of multiple medications increases the risk of adverse drug reactions, which can exacerbate existing health issues in older adults [45]. Studies have consistently shown that polypharmacy is associated with higher rates of morbidity and mortality in this population [46,47]. The nurses in this study emphasized the importance of thorough documentation and regular medication reviews as critical strategies to mitigate these risks. Effective communication and coordination among healthcare providers are essential to managing polypharmacy, as inconsistent documentation and lack of communication can lead to harmful drug interactions [48].

Polypharmacy is a well-documented issue in geriatric care, with research indicating that nearly 50% of older adults take five or more medications concurrently [49]. This practice increases the likelihood of drug–drug interactions, adverse drug reactions, and medication non-adherence [35,50]. Nurses play a crucial role in monitoring and managing these medication regimens to minimize risks. The emphasis on documentation and regular reviews aligns with best practice recommendations for geriatric care, which advocate for medication reconciliation and periodic reviews to ensure that each medication is necessary and appropriate [27].

The management of end-of-life care and advance directives also presents substantial ethical and legal challenges. Nurses often find themselves navigating complex discussions about advance directives, which are critical for ensuring that the patient’s end-of-life wishes are respected [51]. However, these discussions are fraught with difficulties, particularly when family members disagree with the patient’s documented preferences. This discord can lead to ethical dilemmas and potential legal issues if the healthcare team fails to adhere to the patient’s advance directives [52]. The importance of early and clear communication about end-of-life preferences cannot be overstated, as it helps to align the care provided with the patient’s values and legal rights [53].

Advance directives are intended to guide healthcare decisions when patients are no longer able to express their preferences, but their implementation can be challenging in practice [54]. Research indicates that while advance directives are associated with higher satisfaction with end-of-life care and a higher likelihood of dying in the preferred setting, their use remains inconsistent [55,56]. Nurses often encounter situations where advance directives are not available or are not specific enough to guide decision-making. Furthermore, family members may not be aware of or may disagree with the patient’s wishes, leading to conflicts that require careful mediation by healthcare providers [57,58].

The legal implications of these challenges underscore the need for nurses to be well-versed in relevant healthcare laws and regulations [59]. Proper documentation is not only a legal requirement but also a critical component of quality patient care. Incomplete or inaccurate documentation can lead to legal ramifications and adverse patient outcomes [60]. The nurses in this study highlighted the constant pressure to maintain meticulous records, which is essential for legal compliance and protecting patient rights.

Documentation serves multiple purposes in healthcare, including facilitating communication among providers, supporting clinical decision-making, and ensuring legal and regulatory compliance. In the context of multimorbidity, where patients may see multiple providers and take numerous medications, accurate documentation is particularly important [60]. Studies have shown that poor documentation can lead to errors in treatment, delays in care, and increased liability for healthcare providers. Nurses, therefore, must be diligent in their documentation practices, ensuring that all relevant information is recorded accurately and comprehensively [61]. These illustrate the importance of coordinated efforts among healthcare providers to manage polypharmacy. The roles of nurses and doctors are distinct yet complementary in this context. Doctors primarily focus on diagnosing and prescribing medications tailored to specific conditions, while nurses take on the critical role of overseeing the overall medication management. This includes monitoring for adverse effects, ensuring adherence, and facilitating communication between different healthcare providers to create a cohesive and safe treatment plan [27].

By working together, healthcare providers can effectively manage the complexities of polypharmacy, ensuring that patient safety and care quality are maintained. This collaborative approach is essential to navigate the ethical and legal challenges inherent in managing multiple medications for older adults with multimorbidities [62].

Furthermore, the role of nurses as patient advocates is paramount. Advocacy involves not only supporting the rights and autonomy of patients but also addressing systemic issues within the healthcare system that may impede the delivery of high-quality care [63]. This study’s findings indicate that nurses must be proactive in advocating for ethical practices and legal standards within their institutions. This includes pushing for better communication systems, more comprehensive training on ethical and legal issues, and policies that support patient-centered care [64].

Nurse advocacy extends beyond individual patient interactions to include systemic improvements in healthcare delivery [65]. Nurses are in a unique position to identify gaps and inefficiencies in care processes and to advocate for changes that enhance patient outcomes. This can involve lobbying for policy changes, participating in interdisciplinary care teams, and educating patients and families about their rights and options [66]. Advocacy is a critical component of nursing practice, particularly in the context of multimorbidity, where patients often require complex, coordinated care [8].

## 5. Limitations of the Study

This study has several limitations that should be acknowledged. First, the sample size was relatively small, comprising only 15 nurses from the Riyadh region, which may limit the generalizability of the findings. The perspectives and experiences of these nurses might not fully represent those of nurses in other regions or healthcare settings. Additionally, the study relied on self-reported data from semi-structured interviews, which can introduce biases such as recall bias or social desirability bias. Participants may have presented their experiences and practices in a more favorable light, potentially skewing the results. Furthermore, the qualitative nature of the study, while providing in-depth insights, does not allow for the quantification of the prevalence or extent of the identified challenges. Future research could benefit from a larger, more diverse sample and the inclusion of quantitative methods to validate and expand upon these findings.

## 6. Implications of the Study

The findings of this study have significant implications for nursing practice, education, and policy. For nursing practice, the study highlights the need for nurses to receive ongoing training on ethical and legal issues, particularly in the context of caring for older adults with multimorbidities. This training should include strategies for managing informed consent, polypharmacy, and end-of-life care, ensuring that nurses are well equipped to handle these complex situations. For nursing education, incorporating case studies and practical scenarios related to these challenges into curricula can better prepare future nurses for real-world practice. Additionally, healthcare institutions should implement policies that promote effective communication, thorough documentation, and regular medication reviews to enhance patient safety and care quality. On a policy level, there is a need for clearer guidelines and support systems to assist nurses in navigating the ethical and legal complexities of their roles. Advocacy for policies that foster interdisciplinary collaboration and provide resources for ethical reflection and discussion is essential.

## 7. Conclusions

This study provides valuable insights into the ethical and legal challenges faced by nurses in caring for older adults with multimorbidities. The findings highlight the complexities and nuances of nursing practice in this context, emphasizing the importance of balancing patient autonomy with safety, managing polypharmacy, and ensuring effective coordination among healthcare providers. Despite the relatively small sample size of 15 nurses, the study’s purposive sampling and in-depth qualitative approach allowed for rich, detailed data that contributed significantly to our understanding of these challenges. The diversity in participants’ ages, educational backgrounds, and years of experience added depth to the findings, although it also underscores the need for caution in generalizing the results across different healthcare settings.

One key finding of this study is the critical role nurses play in managing polypharmacy, highlighting the importance of vigilant monitoring, patient education, and coordination with multiple healthcare providers. This study elaborates on previous research by providing concrete examples of how nurses navigate these challenges in practice, emphasizing the need for centralized medication records and regular team meetings to ensure patient safety. The study also confirms the ongoing ethical dilemma of respecting patient autonomy while ensuring safety, particularly for patients with cognitive impairments. The findings reinforce the importance of clear communication and documentation to navigate these legal and ethical boundaries effectively.

Furthermore, the study extends existing knowledge by illustrating the practical strategies nurses employ to overcome barriers in communication and coordination, such as the use of telehealth and patient education. These strategies are crucial for enhancing the quality of care and ensuring that patients’ complex needs are met safely and effectively. However, the methodological limitations, including the small sample size and the reliance on self-reported data, must be acknowledged. Future research should aim to include larger, more diverse samples and explore the use of mixed methods to triangulate findings and provide a more comprehensive understanding of these issues.

In conclusion, this study underscores the vital role of nurses in managing the ethical and legal challenges associated with caring for older adults with multimorbidities. By confirming and elaborating on previous findings, it provides practical insights and recommendations for improving nursing practice and policy. Addressing these challenges requires ongoing education, robust communication systems, and supportive institutional policies to empower nurses.

## Figures and Tables

**Table 1:** Participant Characteristics.

Participant ID	Age	Gender	Education	Years of Experience
P1	28	Female	Bachelor’s	5
P2	35	Male	Diploma	12
P3	42	Female	Bachelor’s	18
P4	50	Female	Bachelor’s	25
P5	30	Female	Diploma	7
P6	37	Male	Bachelor’s	13
P7	29	Female	Bachelor’s	6
P8	44	Female	Master’s	20
P9	39	Male	Bachelor’s	15
P10	33	Female	Bachelor’s	9
P11	31	Female	Diploma	8
P12	47	Female	Bachelor’s	22
P13	55	Female	Bachelor’s	30
P14	26	Male	Bachelor’s	2
P15	41	Female	Master’s	17

**Table 2:** Thematic Results: Ethical and Legal Challenges in Caring for Older Adults with Multimorbidities.

Theme	Subtheme	Description
Ethical Dilemmas in Patient Autonomy and Consent	Challenges in Obtaining Informed Consent	Nurses reported difficulties in ensuring patients with cognitive impairments fully understand and consent to treatment.
	Decision-Making with Cognitive Impairment	Nurses faced ethical dilemmas when patients with dementia or other cognitive impairments refused necessary treatments.
	Balancing Autonomy and Safety	Nurses struggled to balance respecting patient autonomy with ensuring patient safety, often navigating ethical gray areas.
Managing Polypharmacy and Patient Safety	Risks of Adverse Drug Reactions	Nurses expressed concerns about the high risks of adverse drug interactions due to multiple medications.
	Documentation and Regular Reviews	Emphasized the need for meticulous documentation and regular reviews to manage and mitigate risks associated with polypharmacy.
	Coordination Among Healthcare Providers	Highlighted the importance of effective communication and coordination among healthcare providers to ensure safe medication management.
End-of-Life Care and Advance Directives	Honoring Advance Directives	Discussed the ethical and legal challenges in respecting patients’ end-of-life wishes, especially when family members disagreed.
	Difficult Conversations	Addressed the necessity and difficulty of having conversations about advance directives and end-of-life care preferences.
	Legal Implications of End-of-Life Decisions	Focused on the legal ramifications of end-of-life care decisions and the importance of clear documentation to avoid legal issues.

## Data Availability

All data are available within the manuscript.
